# Normative volumetric growth modeling of the whole fetal body, placenta, and amniotic fluid for three-dimensional T2-weighted magnetic resonance imaging

**DOI:** 10.1007/s00247-026-06682-3

**Published:** 2026-06-13

**Authors:** Alena Uus, Megan Hall, Charline Bradshaw, Anangsha Kumar, Jordina Aviles Verdera, Sara Neves Silva, Aysha Luis, Hadi Waheed, Pedro Alarcon Gil, Lucilio Cordero-Grande, Jacqueline Matthew, Vanessa Kyriakopoulou, Maria Deprez, Kathleen Colford, Alexia Egloff Collado, Joseph V. Hajnal, Mary Rutherford, Jana Hutter, Lisa Story

**Affiliations:** 1 Research Department of Early Life Imaging, School of Biomedical Engineering and Imaging Sciences, King’s College London, Westminster Bridge Road, London, SE1 7EH UK; 2 Department of Women and Children’s Health, King’s College London, London, UK; 3 Biomedical Image Technologies, ETSI Telecomunicacion, Universidad Politecnica de Madrid and CIBER-BBN, Madrid, Spain; 4 Research Department of Imaging Physics and Engineering, School of Biomedical Engineering and Imaging Sciences, King’s College London, London, UK; 5 Institute of Information Processing, Leibniz University Hannover, Hanover, Germany; 6 Fetal Medicine Unit, Guy’s and St Thomas’ NHS Foundation Trust, London, UK

**Keywords:** Amniotic fluid, Fetus, Growth, Magnetic resonance imaging, Placenta, Volume

## Abstract

**Background:**

Magnetic resonance imaging (MRI)-based volumetry of the fetus, placenta, and amniotic fluid is clinically valuable but rarely used due to labor-intensive manual segmentation of motion-corrupted two-dimensional (2-D) stacks. Existing deep learning approaches are typically limited to single structures and 2-D data, while no robust automated solution exists for whole-uterus volumetry in reconstructed three-dimensional (3-D) MRI, and normative reference ranges are lacking.

**Objective:**

To develop an automated pipeline for whole-uterus volumetry in 3-D T2-weighted fetal MRI and derive normative growth models for fetal, placental, and amniotic fluid volumes.

**Materials and methods:**

Motion-corrupted T2-weighted stacks (0.55–3-T field strength) were reconstructed into 3-D isotropic images using deformable slice-to-volume reconstruction, followed by automated segmentation with a 3-D U-Net. The method was applied to 357 normal-control datasets with confirmed term birth (16–41 weeks gestational age range) to derive quadratic normative growth curves. Performance and clinical utility were further evaluated on 43 independent datasets.

**Results:**

Segmentation was highly accurate (Dice: fetus 0.997, placenta 0.995, amniotic fluid 0.998) with low volume errors (<1%) and minimal manual refinement required in <25% of cases. In the control cohort, fetal and placental volumes increased with gestational age (*P*<0.001), while amniotic fluid followed a quadratic trend. Longitudinal growth rates were 146.6 cc/week (fetus) and 38.8 cc/week (placenta). Preterm pregnancies showed significantly lower fetal and placental volumes (*P*<0.001) and reduced amniotic fluid (*P*<0.01).

**Conclusion:**

This work presents the first automated pipeline for simultaneous whole-uterus volumetry in 3-D fetal MRI and establishes normative growth models across gestation. The approach enables accurate, standardized volumetric assessment and provides a practical tool for detecting abnormal growth patterns in both normal and high-risk pregnancies.

**Graphical abstract:**

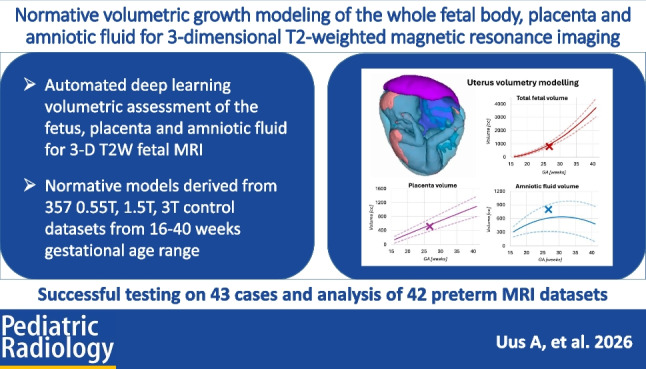

**Supplementary information:**

The online version contains supplementary material available at 10.1007/s00247-026-06682-3.

## Introduction

Fetal magnetic resonance imaging (MRI) is a valuable adjunct to antenatal ultrasound for assessing fetal and placental anatomy, growth, and function. Compared to ultrasound, MRI offers superior soft-tissue contrast, a wider field of view encompassing the entire uterus, and true three-dimensional (3-D) information, improving anatomical assessment and diagnostic confidence [1]. This makes MRI well suited for quantifying volumes of key intrauterine structures - the fetus, placenta, and amniotic fluid, serving as objective markers of fetal growth, placental sufficiency, and overall pregnancy health. These volumes are conventionally obtained from segmentations of MRI stacks.

Quantitative global intrauterine volumetry is clinically informative and important for multiple reasons. Fetal body volume reflects overall fetal size and, when plotted against gestational age (GA)-adjusted centiles, helps identify fetuses that are unusually small or large for gestation, which may indicate fetal growth restriction or macrosomia [2] conditions for which early detection is critical for timely clinical intervention. MRI-derived total fetal volume is also widely used as a denominator for volumetry of individual fetal organs (e.g., brain, lungs) [3–5] and for quantitative flow estimation in fetal cardiac MRI [6], making accurate whole-fetus segmentation essential for many quantitative MRI applications.

Placental volume provides a macroscopic indicator of placental development and functional capacity. Smaller placental volumes are associated with placental insufficiency and conditions such as fetal growth restriction, preeclampsia, and hypertensive disorders, reflecting impaired villous development and reduced fetal growth [7]. Larger placental volumes may occur in gestational diabetes or anemia due to increased metabolic demand [7]. Placental size also correlates strongly with birth weight and neonatal outcomes [8], supporting its value as a complementary biomarker alongside Doppler ultrasound.

Amniotic fluid volume reflects fetal renal and gastrointestinal function, placental health, and membrane integrity [9, 10]. Oligohydramnios (a low amount of amniotic fluid) may result from placental insufficiency or fetal growth restriction through reduced renal perfusion, and is also characteristic of preterm prolabor rupture of membranes (PPROM), where sustained fluid loss can additionally impact lung development. Polyhydramnios (an excess of amniotic fluid) is commonly associated with maternal diabetes or fetal gastrointestinal obstruction (e.g., tracheoesophageal fistula) and is linked to increased perinatal risk. As amniotic fluid is influenced by multiple fetal and placental pathways, its volumetric assessment provides a useful integrated indicator of intrauterine health.

MRI-based 3-D volumetry provides reproducible, objective, and quantitative measurements that overcome the limitations of subjective interpretation, isolated two-dimensional (2-D) metrics, and inherently suboptimal formula-based weight estimates commonly used in ultrasound. Despite these advantages, MRI-based volumetry is rarely performed in routine radiological practice or obstetrics due to high cost, safety concerns, long acquisition times (2–4 min per stack of 2D-slices), and sensitivity to fetal and maternal motion. Most research studies continue to rely on labor-intensive manual segmentation of motion-corrupted balanced steady-state free precession (bSSFP) or T2-weighted (T2W) single-shot turbo spin echo (SSTSE) stacks [11–15]. This approach is slow, operator dependent, and error prone, particularly for large structures such as the amniotic cavity, fetal body, or placenta. Recent deep learning methods offer partial automation, yet most are trained directly on raw motion-corrupted stacks and typically target a single region of interest such as the fetal body [5, 16], placenta [17, 18], or amniotic fluid [19]. Multi-structure approaches [20, 21] remain limited by narrow coverage of gestational age, small training cohorts, and single-protocol datasets. Crucially, slice misalignment and through-plane motion in raw stacks restrict volumetric accuracy, and no existing method enables reliable simultaneous volumetry of fetus, placenta, and amniotic fluid in true 3-D images.

There are also no combined normative growth charts for these three compartments derived specifically from healthy control pregnancies across multiple field strengths. Given the physiological interdependence of fetal, placental, and amniotic volumes, combined assessment has been shown to improve diagnostic certainty [22]. Existing MRI volumetry studies are however heterogeneous, typically focusing on a single structure, restricted GA ranges, or mixed referral populations. Only one recent study reports a normative model for MRI-derived fetal body volume based on 260 healthy fetuses (16–36 weeks GA range) [15]. Comparable normative datasets are lacking for MRI-derived placental or amniotic fluid volumes. Consequently, clinicians lack MRI-based centiles and *z*-scores to determine whether measured volumes fall within expected ranges or to evaluate how the three compartments relate to one another. Establishing normative curves is therefore essential as a robust standardization framework against which individual measurements can be interpreted, which is central to the clinical utility of global intrauterine volumetry. Characterizing cross-compartment and longitudinal relationships then enables early identification of abnormal developmental trajectories. Deformable slice-to-volume registration (DSVR) [23] method addresses these challenges by reconstructing high-resolution isotropic 3-D images from multiple motion-corrupted T2W stacks. This process removes inter-slice misalignment and restores anatomical continuity, enabling more reliable segmentation and volumetry [24]. However, existing 3-D fetal MRI segmentation efforts have focused primarily on the fetal brain or individual fetal organs, and no prior work has provided a solution for combined whole-uterus volumetry including the fetus, placenta, and amniotic fluid in reconstructed 3-D MRI.

This work fills key gaps in MRI-based volumetric assessment of the whole uterus by introducing the first integrated pipeline for automated fetal, placental, and amniotic fluid volumetry in motion-corrected 3-D reconstructed T2-weighted fetal MRI across 0.55–3 -T field strengths. The pipeline provides (1) automated deep learning segmentation of all three structures in 3-D reconstructed volumes; (2) normative growth models derived from 357 confirmed healthy term-control scans covering 16–41 weeks GA range; and (3) a reporting tool that computes centiles and *z*-scores and generates a structured report for clinical interpretation. We further demonstrate the utility of this framework by analyzing patient-specific volumetric trajectories in 95 longitudinal scans from 42 fetuses, and by evaluating deviations in an abnormal preterm birth cohort (*n*=86).

## Materials and methods

### Cohort, image acquisition, and preprocessing

The fetal MRI datasets were acquired at St Thomas’ Hospital, London as part of five ethically approved studies (REC 16/LO/1573, 21/SS/0082, 21/LO/0742, 22/YH/0210, 14/LO/1806, 19/LO/0736). All imaging and data handling procedures adhered to relevant ethical and clinical governance standards, and written informed consent was obtained from all participants.

The study uses T2W SSTSE sequences from fetal MRI datasets acquired at St Thomas’ Hospital, London, across three MRI systems 3-T Philips Achieva (Philips Healthcare, Best, the Netherlands), 1.5-T Philips Ingenia (Philips Healthcare, Best, the Netherlands), and 0.55-T Siemens MAGNETOM Free.Max (Siemens Healthineers, Erlangen, Germany) using protocol variations representative of clinical practice summarized in Table 1.

**Table 1:** MRI acquisition parameters for T2W datasets

Scanner	Datasets	Coil	Echo time (ms)	Resolution (mm)	Thickness/gap (mm)	Stacks
3 T	241	32-channel cardiac	180	1.25×1.25	2.5/–1.5	5–6
1.5 T	122	28-channel torso	80/180	1.25×1.25	2.5/–1.25	6–9
0.55 T	143	6-element flexible+9-element spine	105–106	1.48×1.48	4.5/0	9–12

*T* tesla

The complete dataset (training+analysis) comprises 506 scans from 16–41 weeks’ gestation from the following groups:

- In total, 357 term controls, with confirmed delivery ≥37 weeks and no reported fetal, placental, or maternal abnormalities.
- In total, 86 high-risk cases, consisting of pregnancies with confirmed preterm delivery ≤32 weeks or termination following PPROM.
- In total, 63 additional datasets used exclusively for model training, including cases with various fetal and maternal anomalies, unknown pregnancy outcomes, or outside the main inclusion criteria.

For each case, whole-uterus 3-D reconstruction was performed using deformable slice-to-volume reconstruction (DSVR) [23] implemented in the slice-to-volume reconstruction toolkit (SVRTK) framework (https://github.com/SVRTK/auto-proc-svrtk). This included deep learning (DL) masking of the uterus, semi-automated template stack quality control, and reconstruction to 1.2-mm isotropic resolution. Twenty-eight additional datasets were reconstructed using a more recent deep generative prior DSVR method [25]. In general, the image quality in 0.55-T datasets is inherently suboptimal vs. higher field strengths due to lower resolution and signal-to-noise ratio leading to blurring of finer features.

General inclusion criteria for volumetric analysis were (1) singleton pregnancy; (2) acceptable quality 3-D reconstruction [24] with complete uterine coverage; and (3) sufficient visibility of fetal body, placenta, and amniotic fluid.

The normal-control cohort consists of pregnancies that resulted in healthy term delivery without reported fetal, placental, or maternal structural anomalies. The preterm cohort represents early delivery (≤32 weeks), including cases complicated by PPROM, and therefore reflects clinically relevant intra-uterine pathology. In addition to pregnancy outcomes, we collected available maternal demographic information, including ethnicity, weight, and height, when reported. Birth weight centiles were calculated using neonatal population charts from the Fetal Medicine Foundation [26].

### Automated segmentation

We defined a parcellation protocol for global intrauterine anatomy, comprising four labels: whole fetus (further separated into the fetal head and fetal body), placenta, amniotic fluid, and the umbilical cord set to zero label, which is excluded from volumetry.

**Fig. 1:**
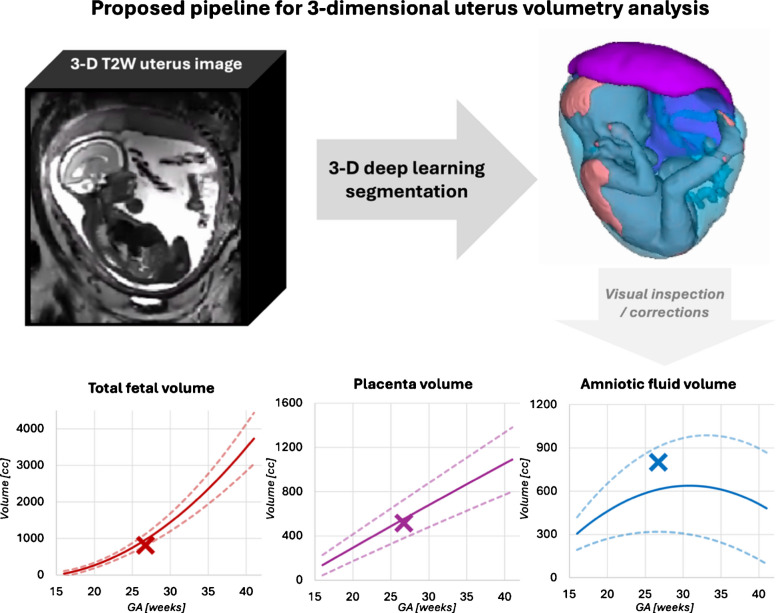
Proposed pipeline for automated volumetric analysis for 3-D T2W fetal MRI. *3-D*, 3-dimensional; *GA*, gestational age; *T2W*, T2-weighted

For automated segmentation (Fig. 1), we trained a deep learning model based on a classical 3D U-Net architecture [27], implemented in the medical open network for artificial intelligence (MONAI) framework [28]. Preprocessing included intensity normalization and resampling with padding to a 256×256×256 grid.

Training was performed using 223 (100 3 T, 30 1.5 T, 93 0.55 T) manually curated 3D-DSVR reconstructions, spanning 17–39 weeks’ gestation and acquired across 0.55-T, 1.5-T, and 3-T MRI systems. The training was performed for 100,000 iterations with standard MONAI augmentations (affine rotations and bias field). The training cohort included normal controls with confirmed delivery at term, high-risk PPROM preterm cases, and fetuses with a range of structural abnormalities, ensuring robustness in diverse anatomy and imaging conditions.

Ground-truth labels were generated through an iterative process combining existing in-house DL models, manual segmentations in ITK-SNAP [29], and multiple rounds of interleaved model training and manual refinement to improve anatomical consistency and boundary accuracy.

Model performance was evaluated on an independent test set of 43 datasets across the range of gestational ages and field strengths (0.55 T, 1.5 T, 3 T) not used during training. During evaluation, each segmentation was inspected in both 2-D slices and 3-D renderings, and quality was graded on a 1–4 scale (1=poor, 2=suboptimal, 3=optimal, 4=good) for every structure. A fetal MRI researcher then manually refined each case to create a reference label, enabling comparison between automated and refined segmentations using Dice score and absolute/relative volume differences.

### Normative growth modeling

The trained model was applied to 357 normal-control 3D-DSVR T2-weighted whole-uterus reconstructions acquired across 0.55-T, 1.5-T, and 3-T systems. All automated labels were visually inspected and, where necessary, manually refined in ITK-SNAP to ensure accurate delineation of the fetus, placenta, and amniotic fluid.

For each case, volumetric measurements (in cubic centimeters) were extracted from the corrected labels. These volumes were then used to construct normative growth models for fetal, placental, and amniotic fluid volumes across gestation. Gestational age trajectories for the 50th, 5th, and 95th centiles were estimated using quadratic regression following established methodology [30].

Statistical analysis in the normal-control cohort was performed using analysis of covariance (ANCOVA) implemented in the *statsmodels* Python library (https://www.statsmodels.org), allowing assessment of associations between volumes, gestational age, and maternal or fetal characteristics.

The resulting normative models were incorporated into a centile calculator (Excel format) and a Python-based reporting tool that generates structured HTML reports including segmentation visualization and centile plots. In addition, estimated fetal weight was computed from fetal volume based on formula defined by Baker et al. (*EFW*_Baker_(kg)=1.031*·V*_fetus_+0.12) [31]. All automated segmentation components, centile calculators, and reporting scripts are publicly available online at the auto-SVRTK repository (https://github.com/SVRTK/auto-proc-svrtk).

### Volumetric analysis of term versus preterm cohorts

To assess the clinical utility of the segmentation pipeline and normative models, we performed a comparative analysis between the 86 preterm cases (delivery ≤32 weeks, including PPROM) and the normal-control cohort. All preterm datasets were segmented using the same automated network and underwent visual quality review, with manual refinements applied when necessary to ensure consistency across groups.

Volumetric comparisons were conducted using both absolute volumes and GA–adjusted centiles, allowing evaluation of whether fetuses destined for preterm birth deviated from expected growth patterns at the time of MRI. Statistical analysis was performed using ANCOVA, correcting for gestational age at MRI.

## Results

### Automated segmentation

**Fig. 2:**
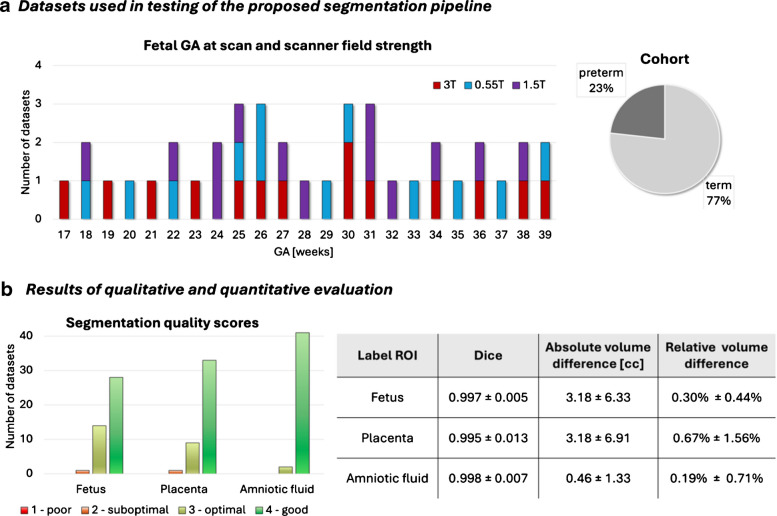
**a** Exemplary fetal MRI datasets used in testing of the proposed segmentation pipeline. **b** Qualitative and quantitative results of testing. *GA*, gestational age; *ROI*, region of interest; *T*, tesla

**Fig. 3:**
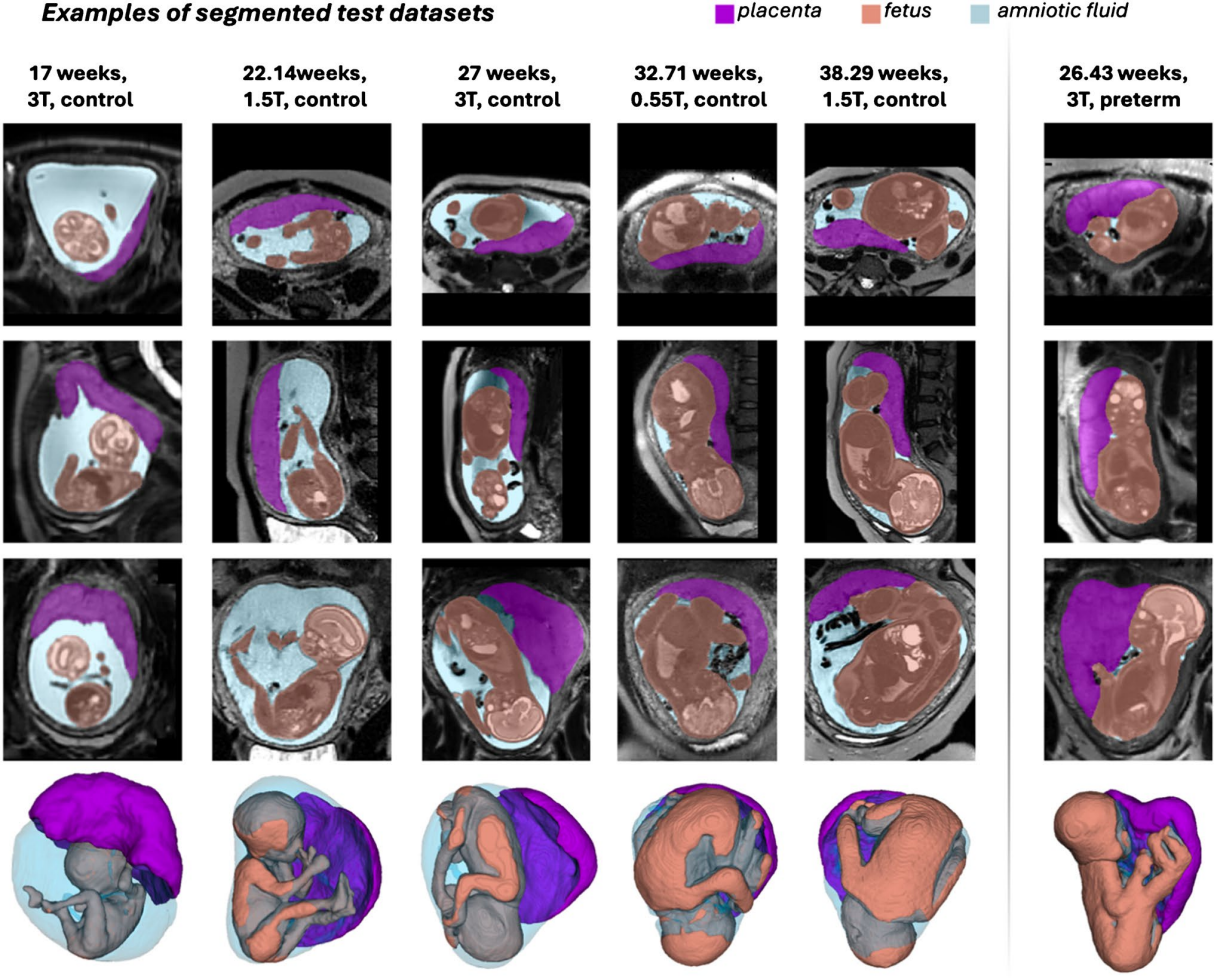
Examples of 3-D segmentations for test cases from different gestational ages, scanner field strengths, and cohorts. *T*, tesla

The trained segmentation model was evaluated on 43 independent datasets spanning all three field strengths, a wide gestational age range, varied fetal and placental positions, and both term and preterm pregnancies (Fig. 2). Example segmentations at 17–38 weeks’ gestation are shown in Fig. 3. Across this range, the 3-D labels showed smooth, anatomically plausible boundaries and realistic appearances for all structures. The qualitative scores and quantitative results are summarized in Fig. 2.

Overall, segmentation quality was high, with most structures receiving good or excellent ratings. The average quality scores were 3.63±0.54 for the fetus, 3.74±0.49 for the placenta, and 3.95±0.21 for amniotic fluid. Only one fetal and one placental segmentation were scored as suboptimal. This was consistent with the quantitative metrics, which showed high Dice similarity and low relative volumetric error. Most refinements were required at the placenta–myometrium interface, where contrast can be limited and placental morphology irregular, and around fetal limbs, which may be difficult to distinguish from the umbilical cord - particularly in PPROM cases with reduced amniotic fluid and in 0.55-T datasets where image quality is lower.

### Normative growth modeling

**Fig. 4:**
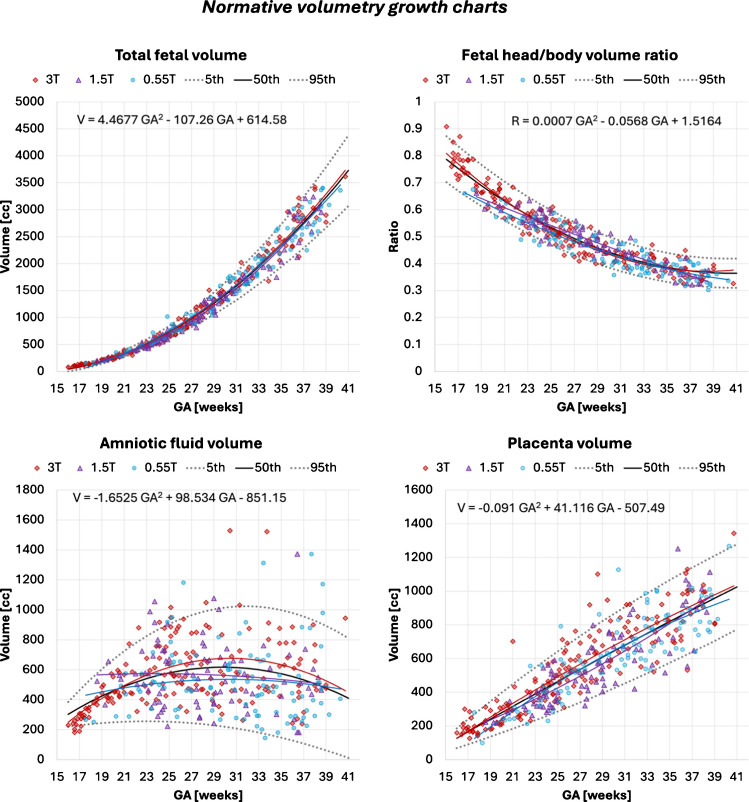
Normative growth charts created from automated volumetry outputs for 357 term-control subjects (*n*=102 at 0.55 T, *n*=88 at 1.5 T, *n*=167 at 3 T) with centiles. *GA*, gestational age; *T*, tesla

Summary characteristics of the normal-control cohort are shown in Supplementary Fig. S1. Automated segmentation outputs for all 357 control datasets were visually reviewed. Most were immediately suitable for volumetric analysis; minor manual refinement was required in 87 cases (<25% of all datasets), predominantly at the placenta–myometrium interface where contrast is limited and around fetal limbs. These refinements (e.g., part of umbilical cord segmented as fetal limbs, parts of placenta under-segmented) were fast (typically <2 min per case) and had no meaningful impact on the final growth models. *z*-scores and centiles were subsequently computed for all volumetric measures. Figure 4 presents the derived quadratic gestational age growth curves (5th, 50th, 95th centiles) for fetal, placental, and amniotic fluid volumes, as well as the fetal head–to–body volume ratio.

The fetal volume showed the expected strong positive association with gestational age (*P*<0.001), with ranges consistent with previously reported MRI norms [12, 15].

Variability increased after approximately 30 weeks, reflecting greater anatomical heterogeneity and expected biological dispersion. The fetal head–body ratio decreased with gestational age (*P*<0.001), following known patterns of proportional body growth and relative reduction in head dominance [32].

The placental volume also increased significantly with GA (*P*<0.001) within similar ranges to reported earlier works [12, 14]. It showed positive associations with maternal height and maternal weight (both *P*<0.01) after correction for GA. Placental volume and placental centiles additionally correlated with fetal volume and fetal volume centiles (*P*<0.001). This aligns with the well-established relationship between placental mass and fetal growth, as larger placentas typically reflect greater villous surface area and better uteroplacental perfusion [7, 8].

The amniotic fluid volume displayed substantial inter-subject variance across gestation (in agreement with earlier works [12]) but followed a significant quadratic pattern (*P*<0.001), with a mid-gestation peak and a decline toward term - consistent with known physiological mechanisms of fetal swallowing, renal output, and placental fluid transfer [33]. Higher amniotic fluid volumes and centiles were positively correlated with placental volume and placental centiles (*P*<0.01), reflecting the shared dependencies on placental perfusion and fetal health.

**Fig. 5:**
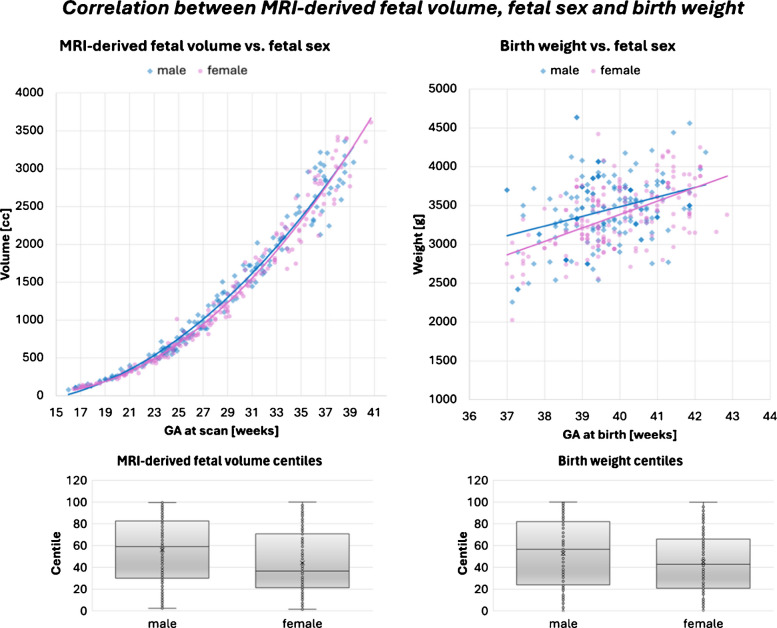
Correlation between MRI-derived fetal volume, fetal sex, and birth weight (male - *blue*, female - *pink*). *GA*, gestational age

After correcting for gestational age at delivery, birth weight was strongly correlated with fetal volume, placental volume, and head–body ratio centiles (*P*<0.001). Higher fetal and placental volumes and lower head–body ratios (which reflect proportionately larger fetal bodies) were associated with higher birth weight. Amniotic fluid centiles were also associated with birth weight (*P*<0.001). Additionally, fetal volume centiles differed between sexes, with higher volumes in male fetuses, matching known sex differences in fetal growth trajectories and birth weight distribution (Fig. 5) [34].

Although general volumetric trends overlapped between scanners, ANCOVA revealed significant field-strength effects on fetal volume between 1.5 T and 3 T (*P*<0.001) and placenta volume between 1.5 T and 3 T (*P*<0.01) with slightly smaller values in the 1.5-T cohort. These differences are likely driven by unequal GA distributions, a larger proportion of cases at 3 T, and other protocol-related factors such as resolution rather than true biological variation. This underlines the importance of harmonized acquisition protocols and careful matching of inclusion criteria when deriving normative volumetric models across multiple field strengths.

**Fig. 6:**
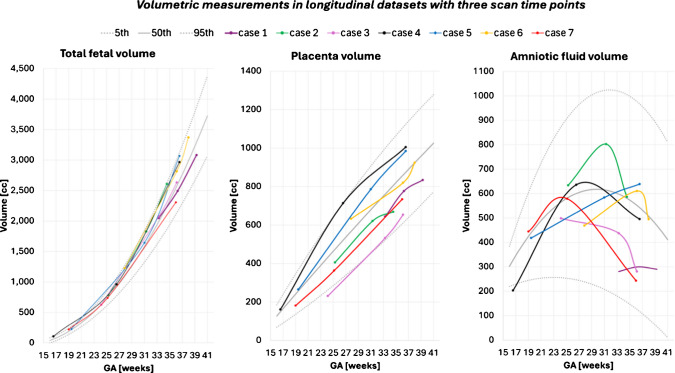
Examples of volumetric changes in longitudinal datasets of 7 subjects scanned at three scan GA time points. *GA*, gestational age

To assess the correlation of patient-specific growth trajectories with the normative models, we performed volumetric analysis of longitudinal datasets of 42 fetuses with multiple GA time points (95 scans in total) from the control term cohort. Figure 6 shows examples of changes in seven cases with three time points. The fetal volume demonstrates steady growth with gestation and follows the growth model trend (146.6±39.4 cc/week growth rate slope). Placenta volume is also increasing throughout pregnancy similarly to the normative model trajectory (38.8±14.7 cc/week growth rate slope). The changes in amniotic fluid volume show high variability between the cases (3.3±35.5 cc/week growth rate slope) with a decreasing trend toward late gestation, which agrees with the expected physiological pattern with peak in mid-gestation and then a gradual decrease toward term [33]. Across fetuses, the individual growth rates of fetal and placental volume were only weakly correlated (*r*≈0.12). Interestingly, fetal growth rate showed a moderate negative correlation with the amniotic fluid slope (*r*≈0.44), suggesting that cases with rapidly increasing fetal volume tended to show stable or declining amniotic fluid volumes.

Overall, these findings demonstrate the validity and clinical relevance of the generated normative volumetry models, while highlighting the need for future work incorporating detailed demographic factors, abnormal cases, and outlier centiles to support personalized growth prediction and improved diagnostic stratification.

### Volumetric analysis of term vs. preterm cohorts

**Fig. 7:**
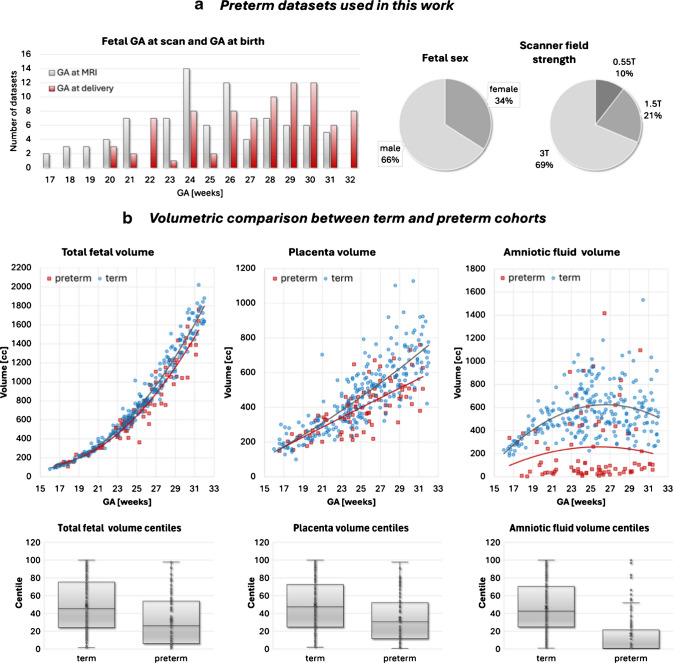
Term vs. preterm cohort analysis. **a** Summary information on the abnormal preterm cohort (*n*=86) used in a feasibility sub-study. **b** Volumetric comparison between term (*n*=252) and preterm (*n*=86) cohorts. *GA*, gestational age; *MRI*, magnetic resonance imaging; *T*, tesla

To assess the feasibility and clinical utility of the proposed automated volumetry pipeline, we applied it to a heterogeneous abnormal cohort of 86 preterm datasets that included a mix of pregnancies with PPROM and pregnancies with intact membranes, all resulting in delivery ≤32 weeks GA, and compared these with 252 term-born controls scanned at ≤32 weeks GA. The preterm group (Fig. 7) encompassed a range of clinical indications and pregnancy complications, including spontaneous preterm birth, preeclampsia requiring elective delivery, and PPROM, as well as PPROM cases electing for termination. These participants were recruited on the basis of a high predicted risk of preterm birth and subsequently delivered at <32 weeks GA.

All preterm datasets were segmented automatically, followed by visual review. Manual refinement was required in 35 cases, mainly in the placenta and fetal body regions. This was typically due to reduced soft-tissue visibility and marked amniotic fluid loss in PPROM, and refinements were brief (<5 min per case). These findings highlight the importance of further model retraining for atypical anatomy and underline the need for automated quality-control methods within future versions of the pipeline.

As shown in Fig. 7, fetal volumes in the preterm cohort show a broad spread but a significantly lower trendline compared with the term controls (*P*<0.001), reflected in lower fetal volume centiles (*P*<0.001) in agreement with earlier studies [35]. Placental volumes and placental centiles were likewise markedly lower in preterm pregnancies (*P*<0.001), consistent with impaired placental development or compromised uteroplacental perfusion described in preterm birth and hypertensive disorders [7].

Amniotic fluid volumes in the preterm cohort were also significantly reduced, particularly in PPROM cases, where membrane rupture results in fluid loss. This trend was reflected in both absolute volume (*P*<0.01) and centiles (*P*<0.001).

Examples of automatically generated.html volumetry reports for PPROM cases are shown in Supplementary Fig. S2. In both examples, amniotic fluid centiles were <5th centile. In one case, both fetal and placental volumes were <5th centile, while in the other case, fetal and placental volumes remained within normal ranges despite severe oligohydramnios. The report format and centile-based interpretation were reviewed by fetal MRI clinicians and considered clear, clinically meaningful, and useful for integration into routine reporting workflows.

Together, these results demonstrate that the automated volumetry pipeline can capture meaningful physiological differences between normal and high-risk pregnancies and support the feasibility of its application in clinical and research settings. They also illustrate the potential for this tool to serve as a baseline framework for further refinement and retraining toward specific clinical scenarios and pathology-focused models.

## Discussion

This study presents the first integrated framework for automated volumetry of the fetus, placenta, and amniotic fluid in motion-corrected 3-D reconstructed T2W fetal MRI across 0.55-T, 1.5-T, and 3-T acquisition protocols. Using deformable slice-to-volume reconstruction and a 3-D U-Net, we derived normative volumetric growth charts from a large and well-defined cohort of 357 confirmed healthy term-control pregnancies scanned between 16–41 weeks GA. This directly addresses longstanding limitations of prior MRI volumetry, which relied on labor-intensive manual segmentation of 2-D motion-corrupted stacks with DL models predominantly trained to segment only a single region of interest. Importantly, there is also lack of combined true normative MRI volumetry models for the fetus, placenta, or amniotic fluid generated from confirmed healthy control cohorts, despite the clinical need for reliable reference curves for *z*-scores and abnormality detection.

The segmentation model performed robustly across field strengths and gestational ages, with only minor manual refinements required - mostly at the placenta–myometrium interface, a known area of low contrast in fetal MRI. Performance across heterogeneous acquisition protocols supports the generalizability of the approach and its utility in both retrospective and prospective datasets.

The derived growth curves reproduced well-established physiological patterns. Fetal volume increased steadily with widening variance toward term, consistent with ultrasound and MRI literature [15]. Placental volume increased throughout gestation, reflecting villous maturation and vascular expansion, and showed positive associations with maternal height, weight, and birth weight - relationships that align with known correlations between placental mass, uteroplacental capacity, and neonatal size. Amniotic fluid volumes displayed the expected high inter-subject variability and characteristic mid-gestation peak with a decline toward late pregnancy, reflecting the changing balance between fetal urine production, swallowing, and intramembranous absorption.

MRI-derived volumetry also demonstrated meaningful correlations with birth weight. Higher fetal and placental volumes, and lower fetal head–body ratios, were associated with higher birth weight after GA correction. Sex-related differences in total fetal volume (larger in males) further matched established biological patterns.

Analysis of 95 longitudinal scans from 42 term-control fetuses showed that fetal and placental growth trajectories followed the normative curves, whereas amniotic fluid slopes were more variable - consistent with the dynamic physiology of amniotic fluid regulation. These findings highlight the potential of MRI volumetry for personalized growth assessment when ultrasound is inconclusive or insufficient.

Application to 86 preterm pregnancies delivered at ≤32 weeks demonstrated significantly lower fetal, placental, and amniotic fluid volumes and centiles relative to matched term controls. These findings align with known impairments in placental function, fetal growth, and membrane integrity in PPROM, preeclampsia, and spontaneous preterm birth. Mild refinements were occasionally required in cases with severe fluid loss, but automated segmentation remained feasible across the cohort, indicating real-world clinical applicability.

Overall, our findings demonstrate the value of automated whole-uterus volumetry for detecting deviations from expected growth trajectories in both cross-sectional and longitudinal settings. This pipeline provides a practical foundation for quantitative fetal and placental MRI.

In terms of limitations, segmentation accuracy remains partly dependent on DSVR reconstruction quality and local image contrast. The placenta–myometrium interface and fetal extremities continue to present challenges, particularly in PPROM cases with markedly reduced amniotic fluid. Future work could incorporate targeted augmentation, contrast-aware architectures, or attention-based models to reduce the need for manual refinement, alongside automated quality-control scoring to improve reliability. The normative models were derived from healthy pregnancies with confirmed delivery at term, but several relevant biological and demographic modifiers - such as fetal sex, maternal age, height, weight, ethnicity, and general health or fitness - were not explicitly incorporated, despite their known influence on fetal and placental size. Future extensions will focus on demographic-adjusted normative curves and mixed-effects, Gaussian-process modelling, or other fitting approaches such as the Gompertz model, which may better accommodate the nonlinearity of growth trajectories. Broader inclusion of ethnically and socioeconomically diverse cohorts will also be essential for improving geographic and population-level generalizability.

The current pipeline focuses on global intrauterine structures. Extending the framework to additional fetal organs (e.g., the lungs, liver, brain) [36, 37] would support organ-specific volumetry pipelines, which frequently rely on total fetal volume for normalization. Further integration of placental surface area estimation, automated placental site characterization, umbilical cord insertion localization, and fetal lie assessment could enrich the utility of whole-uterus analysis.

Finally, multi-center validation across vendors, field strengths, and acquisition protocols will be important to establish generalizability and accelerate adoption in routine clinical workflows. Complementary studies incorporating paired MRI–ultrasound volumetry, for both whole-uterus and organ-specific measurements, will offer insight into cross-modality reproducibility and help define how automated MRI volumetry can best complement established ultrasound-based assessment.

## Conclusion

We present the first automated framework for simultaneous fetal, placental, and amniotic fluid volumetry in motion-corrected 3-D reconstructed T2-weighted fetal MRI, together with the first combined normative volumetric growth models derived from a large cohort of confirmed healthy term-control pregnancies at 0.55–3-T scanner field strengths. The pipeline provides efficient, standardized volumetric assessment across a wide range of MRI field strengths, supports individualized interpretation through *z*-scores and centiles, and performs reliably in both normal and high-risk pregnancies. Demonstrated applicability in longitudinal and preterm cohorts underscores its potential for integration into routine fetal MRI assessment and future quantitative fetal and placental research.

## Supplementary information

Below is the link to the electronic supplementary material.

ESM 1 (PDF 1.09 MB)

## Data Availability

The generated normative models and code for processing are publicly available online at auto-SVRTK repository: https://github.com/SVRTK/auto-proc-svrtk. The individual fetal MRI datasets used for this study are not publicly available due to ethics regulations. For more information, please contact Jana Hutter jana.hutter@kcl.ac.uk.
