# Supplementary material for: Normative volumetric growth modeling of the whole fetal body, placenta, and amniotic fluid for three-dimensional T2-weighted magnetic resonance imaging
Source: Pediatr Radiol. 2026 Jun 13;56(8):1789–802. doi: 10.1007/s00247-026-06682-3 (PMC13407711; doi:10.1007/s00247-026-06682-3)
Supplement: Supplementary file 1 — (PDF 1.09 MB) [file 247_2026_6682_MOESM1_ESM.pdf]

Supplementary Materials

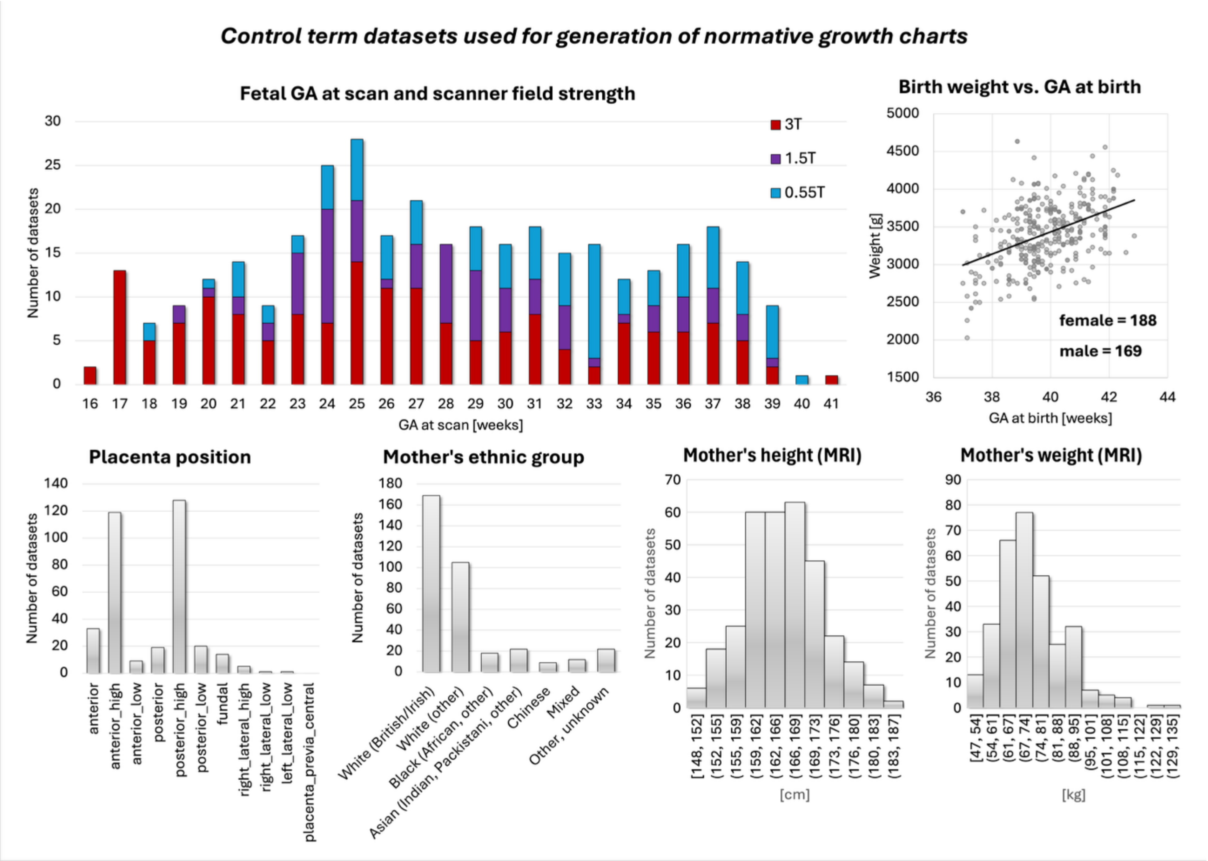

Fig. S1 Summary information on the control term cohort (n=357) used for generation of the normative growth charts. *GA* gestational age, *MRI* magnetic resonance imaging, *T* tesla

Examples of generated .html volumetry reports for preterm datasets

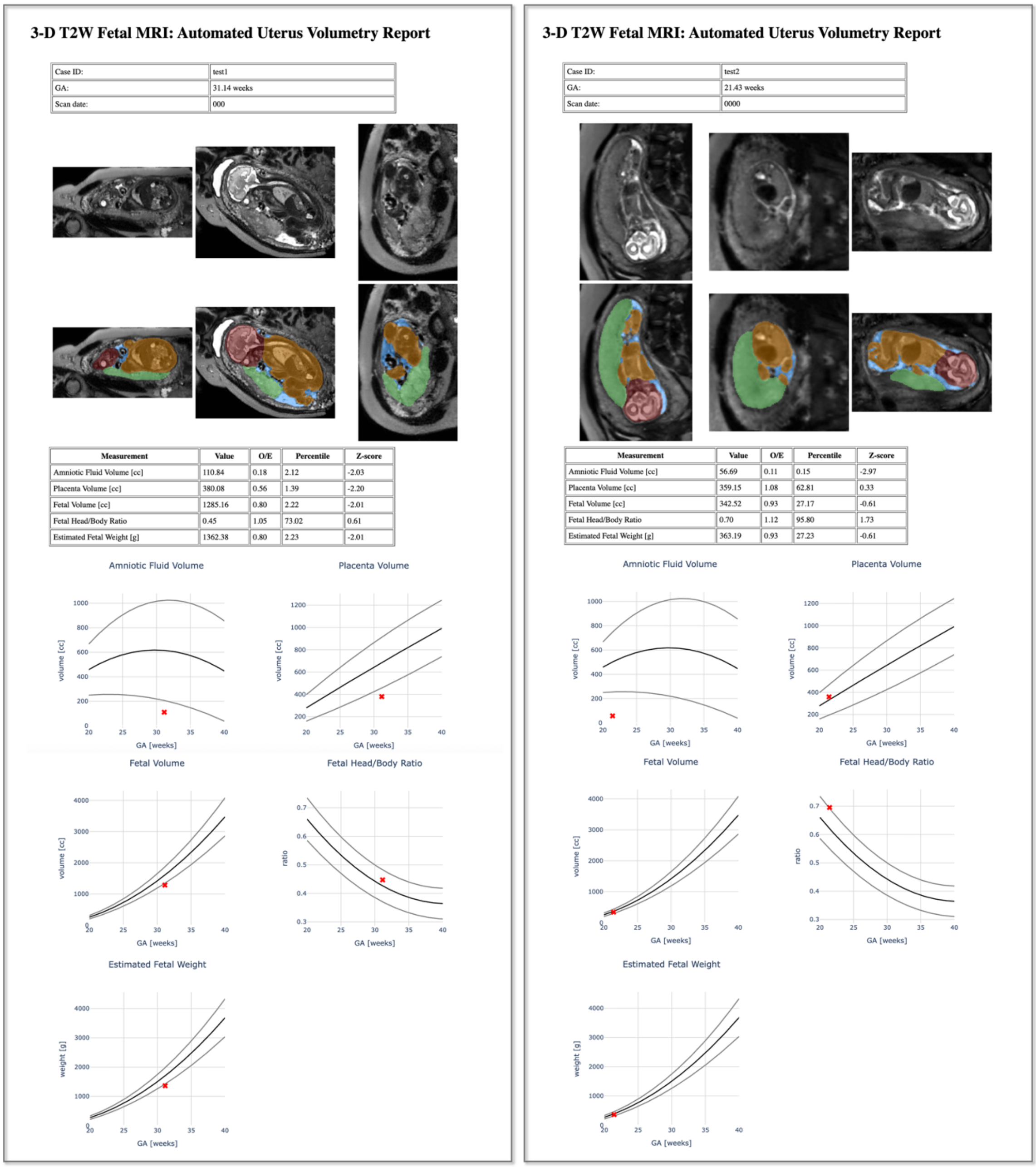

3-D T2W Fetal MRI: Automated Uterus Volumetry Report

Case ID:	test1
GA:	31.14 weeks
Scan date:	000

Measurement	Value	O/E	Percentile	Z-score
Amniotic Fluid Volume [cc]	110.84	0.18	2.12	-2.03
Placenta Volume [cc]	380.08	0.56	1.39	-2.20
Fetal Volume [cc]	1285.16	0.80	2.22	-2.01
Fetal Head/Body Ratio	0.45	1.05	73.02	0.61
Estimated Fetal Weight [g]	1362.38	0.80	2.23	-2.01

3-D T2W Fetal MRI: Automated Uterus Volumetry Report

Case ID:	test2
GA:	21.43 weeks
Scan date:	0000

Measurement	Value	O/E	Percentile	Z-score
Amniotic Fluid Volume [cc]	56.69	0.11	0.15	-2.97
Placenta Volume [cc]	359.15	1.08	62.81	0.33
Fetal Volume [cc]	342.52	0.93	27.17	-0.61
Fetal Head/Body Ratio	0.70	1.12	95.80	1.73
Estimated Fetal Weight [g]	363.19	0.93	27.23	-0.61

Fig. S2 Examples of automated reports generated as .html files. *3-D* 3-dimensional, *GA* gestational age, *MRI* magnetic resonance imaging, *O/E* observed-to-expected, *T2W* T2-weighted
